# Visual and Anatomical Outcomes of Intravitreal Aflibercept for Treatment-Resistant Neovascular Age-Related Macular Degeneration

**DOI:** 10.1155/2014/273754

**Published:** 2014-05-07

**Authors:** Magda Gharbiya, Ludovico Iannetti, Francesco Parisi, Umberto De Vico, Maria Laura Mungo, Marco Marenco

**Affiliations:** ^1^Sapienza University of Rome, Policlinico Umberto I Hospital, 155 Viale del Policlinico, 00161 Rome, Italy; ^2^Casa di Cura Privata Villa Benedetta, 65 Circonvallazione Cornelia, 00165 Rome, Italy; ^3^Casa di Cura Privata Villa Margherita, 48 Viale di Villa Massimo, 00161 Rome, Italy

## Abstract

A retrospective chart review of patients with persistent subretinal and/or intraretinal fluid, despite previous treatment with intravitreal ranibizumab (0.5 mg), who were switched to aflibercept injections, was performed. Treatment was three monthly aflibercept (2 mg) injections followed by dosing on pro re nata basis. Main outcome measures included changes in best corrected visual acuity (BCVA), 1 mm central subfield (CSF) retinal thickness, the height of the pigment epithelial detachment (PED), and subfoveal choroidal thickness on optical coherence tomography at 6 months. Thirty-one eyes of 30 patients were analyzed. The mean number of injections before aflibercept conversion was 34.4 ± 11.9. After an average of 4.5 aflibercept injections (range 3 to 6) over 6 months, no significant change in BCVA was observed (*P* > 0.05). Compared with baseline, there was a significant reduction of the CSF retinal thickness (449 ± 179 versus 269 ± 145 **μ**m, *P* < 0.001), maximum PED height (262 ± 134 versus 183 ± 100 **μ**m, *P* < 0.001), and choroidal thickness (192 ± 67 versus 167 ± 51 **μ**m, *P* < 0.01). Stable visual acuity and anatomical improvement were obtained for up to 6 months after aflibercept conversion. However, choroidal thinning related to treatment was observed.

## 1. Introduction

Since 2004, intravitreal antivascular endothelial growth factor (VEGF) therapy has become the standard treatment for neovascular age-related degeneration (AMD) and has radically changed the management and the clinical prognosis of this disease. Results from large clinical trials have demonstrated that treatment with ranibizumab (Lucentis; Genetech/Roche) or bevacizumab (Avastin; Genetech/Roche) stabilizes vision in more than 90% of eyes and up to a third improve vision by 15 letters or more [1–3]. However, as the long-term experience using these agents increased, it has been realized that there are cases that do not respond completely and, despite continuous treatment with either ranibizumab or bevacizumab, have persistent fluid on optical coherence tomography (OCT) [4, 5]. This long-term effect of anti-VEGF therapy may be attributed either to a suboptimal response related to the intrinsic characteristics of the neovascular membrane or to the development of tachyphylaxy/tolerance phenomena, over time. Whatever the underlying mechanism, this particular group of patients represents a growing concern for the treating clinicians. One of the options in this setting is to switch to a similar drug with different properties. In November 2011, a new anti-VEGF drug, aflibercept (VEGF Trap-Eye, Eylea, Regeneron/Bayer), a recombinant fusion protein binding the VEGF, has been approved by the US Food and Drug Administration for the treatment of exudative AMD. This soluble decoy receptor is composed of components of both VEGF receptor 1 (VEGFR1) and 2 (VEGFR2) fused to the Fc region of human IgG1. Compared with ranibizumab and bevacizumab, which only bind to VEGF-A, aflibercept also binds to VEGF-B and placental growth factor (PIGF). Both VEGF-B and PIGF have been implicated in the neovascularization process of AMD. Pharmacokinetic studies have shown that aflibercept has a stronger binding affinity for VEGF-A and a higher trough binding activity than both ranibizumab and bevacizumab; this theoretically should increase its efficacy in neutralizing VEGF and prolong its duration of action [6]. Aflibercept efficacy in patients with newly diagnosed neovascular AMD was demonstrated in two parallel phase III trials [7]. Results from these trials showed that aflibercept achieves similar outcomes compared with ranibizumab and, after 3 monthly initial doses, can be administered every 2 months. Given the efficacy of aflibercept in treatment-naive eyes and its specific molecular profile, it is possible that improved outcomes may also be achievable in patients with refractory neovascular AMD previously treated with bevacizumab or ranibizumab. Indeed, recent studies showed that aflibercept may be effective in this setting. However, the majority of these studies are characterized by combining data of patients with different follow-up times, nonstandard treatment protocols, nonstandardized follow-up intervals, and different treatment regimen [8–15].

The purpose of this retrospective, noncomparative study was to evaluate the 6-month functional and anatomical outcomes of intravitreal aflibercept for refractory neovascular AMD. Choroidal thickness changes related to treatment were also investigated.

## 2. Patients and Methods

We reviewed the medical records of patients with treatment-resistant neovascular AMD, who were converted to on-label aflibercept injections between January 2013 and June 2013. The study was conducted in a multicenter private practice setting in Rome, Italy. All patients gave written informed consent to aflibercept conversion. The patients were all aware that the treatment protocol was modified from that recommended by the pharmaceutical company. From this cohort, patients who received consecutive injections of aflibercept and who met the inclusion and exclusion criteria were finally selected.

Inclusion criteria were as follows*:* (1) persistent intraretinal or subretinal fluid with or without pigment epithelial detachment (PED) at the initiation of aflibercept; (2) at least six consecutive monthly injections with ranibizumab before aflibercept initiation; (3) the interval between the last ranibizumab and the first aflibercept had to be not less than 4 weeks and not exceeding 6 weeks; (4) eligible eyes could have been treated with intravitreal bevacizumab; (5) at least 6 months of follow-up on a monthly basis. Patients were excluded if they had (1) prior treatment with photodynamic therapy; (2) a diagnosis of retinal angiomatous proliferation or idiopathic polypoidal choroidal vasculopathy; (3) any ocular disease that could affect the best-corrected visual acuity (BCVA); (4) a history of intraocular surgery except for uncomplicated phacoemulsification performed within the preceding 6 months; and (5) any systemic condition contraindicating the use of intravitreal anti-VEGF agents.

The following data were collected from each chart: patient age, gender, diagnosis, past ocular history, including dates and types of intravitreal injections, the interval between the last ranibizumab and the first intravitreal aflibercept injection, and coexisting ocular conditions.

Before and after aflibercept initiation, all patients had to have a complete ophthalmic examination, including BCVA measurement, optical coherence tomography (OCT), and fundus photography. BCVA was measured using the standardized, 70-letter Early Treatment Diabetic Retinopathy Study (ETDRS) chart (Chart “R,” Precision Vision, La Salle, IL, USA) at 4 meters distance. OCT images were obtained using the Spectralis OCT (Spectralis Family Acquisition Module, V 5.1.6.0; Heidelberg Engineering, Heidelberg, Germany), following a standardized protocol. Active eye-tracking (TruTrack) and automatic follow-up scan (AutoRescan) were used to enable point-to-point correspondence between consecutive follow-up scans. A 20°, 6-radial line scan protocol, centered on the fovea, with at least 50 frames averaged for each scan, was performed for each eye. On each scan of the radial protocol, we evaluated the presence or absence of intraretinal fluid (IRF), subretinal fluid (SRF), and PED. PEDs were classified into three categories based on the reflectivity of the sub-RPE material on OCT: hollow (primarily hyporeflective), solid (primarily hyperreflective), and mixed (heterogeneous signal) [16]. The maximum height of a PED, defined as the distance between the outer border of Bruch membrane and the inner border of the RPE, was measured anywhere on the fovea scan, using the digital caliper tool. We also evaluated the status (disrupted or complete) at baseline of the foveal inner/outer segment (IS/OS) junction line along a 1-mm-diameter area centered on the fovea. A macular thickness map was obtained for each eye, using the raster horizontal 20° × 15°, 19-line scan protocol, centered on the fovea, with at least 25 frames averaged for each scan. The automated segmentation of scans covering the central 1-mm subfield was manually corrected to ensure proper segmentation of the inner limiting membrane and the retinal pigment epithelium (RPE) band. 1-mm central subfield (CSF) retinal thickness, measured automatically by the OCT software, was then recorded for each eye. SD-OCT images of the choroid were obtained by enhanced depth imaging modality. Two high-quality 30° horizontal and vertical line scans through the fovea with 90 to 100 frames averaged for each scan were obtained for each eye and the image with the best visualization of the border between the choroid and sclera was used. Choroidal thickness was measured using the digital caliper tool. The choroid was defined as the layer between the base of the RPE and the hyperreflective line or margin corresponding to the chorioscleral interface. Subfoveal choroidal thickness from the horizontal and vertical line scans was measured, and values were averaged. Two independent observers took each manual measurement, and the average of both values was used for data analysis.

All patients received a loading dose of three monthly aflibercept injections (2 mg/0.05 mL). Follow-up examinations were given monthly. Retreatment with a single aflibercept injection was performed according to any of the following criteria: (1) visual acuity loss of at least five letters with OCT evidence of fluid in the macula; (2) persistent or recurrent intraretinal or subretinal fluid on OCT; (3) new subretinal hemorrhage from the CNV.

### 2.1. Statistical Analysis

Statistical analysis was performed with the SPSS for Windows (V 17.0, SPSS). All variables were tested for normal distribution using the Kolmogorov-Smirnov test. Continuous variables were compared using paired *t*-test. Categorical variables were compared using McNemar's test. Follow-up data were compared with baseline. Bivariate relationships were examined using the Pearson correlation analysis. Data are reported as mean values ± standard deviation. *P* values <0.05 were considered significant.

## 3. Results 

Forty-two eyes of 39 patients were treated with intravitreal aflibercept for treatment-resistant neovascular AMD during the study period. Nine patients were excluded as they failed to meet the inclusion criteria. The main cause for exclusion was an incomplete follow-up. Finally, there were 31 eyes of 30 patients included in the study. Baseline characteristics of the patients at the time of aflibercept initiation are summarized in Table 1. All patients were previously treated with a loading dose of 3 monthly anti-VEGFs (bevacizumab or ranibizumab) injections followed by a pro re nata (PRN) protocol. Twenty-one eyes were treated with ranibizumab alone (0.5 mg/0.05 mL), and 10 eyes were switched from bevacizumab (1.25 mg/0.05 mL) to ranibizumab during the course of their treatment for neovascular AMD. The length of previous anti-VEGF treatment was 41.3 ± 14.2 months (range, 15 to 58 months). The study eyes had received an average of 34.4 ± 11.9 injections (range, 15 to 50) prior to treatment with aflibercept, with a mean injection interval of 5.1 ± 0.5 weeks (range, 4 to 6). Conversion to aflibercept took place on average 4.9 ± 0.8 weeks (range, 4 to 6 weeks) after the last ranibizumab injection. The mean number of aflibercept injections during the 6-month study period was 4.5 ± 1.3 (range, 3 to 6), with a mean injection interval of 5.8 ± 1.7 weeks (range, 4 to 8 weeks). Five (16%) of the 31 treated eyes received only the loading dose of 3 aflibercept injections.

### 3.1. Visual Outcomes

**Figure 1:**
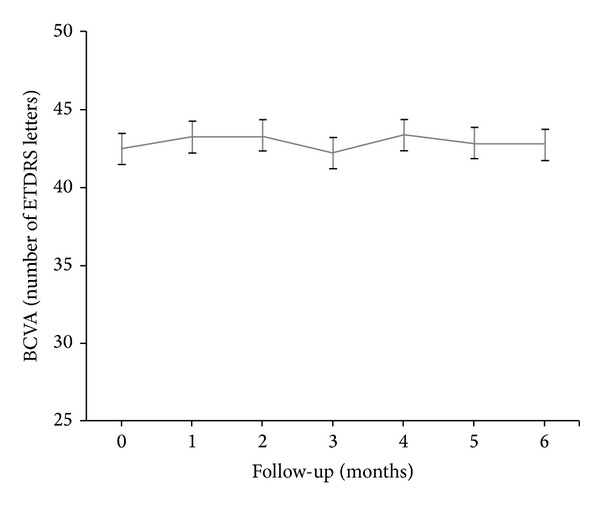
Visual results over time after aflibercept conversion. BCVA: best-corrected visual acuity; ETDRS: early treatment diabetic retinopathy study.

Visual results over time are shown in Table 2. The mean BCVA before aflibercept initiation was 42.5 ± 12.5 ETDRS letters (range, 22 to 60 letters). After 3 aflibercept injections, the average BCVA was 42.3 ± 10.5 letters (range, 25 to 60 letters) (*P* > 0.05) and after 6 months it was 42.8 ± 10.0 letters (range, 27 to 60 letters) (*P* > 0.05), which was not significantly changed compared with baseline (Figure 1). Mean BCVA change at 6 months was +0.3 ± 4.3 letters. At 6 months after aflibercept initiation, 8 eyes (26%) improved by 5 letters or more, 19 eyes (61%) remained stable, and 4 eyes (13%) lost 5 letters or more. No treated eyes experienced a BCVA change of more than 10 letters. BCVA outcome at 6 months was strongly correlated with baseline BCVA (*r* = 0.98, *P* < 0.0001) and with the IS/OS status before aflibercept initiation (*r* = 0.89, *P* < 0.01). In addition, BCVA outcome was negatively influenced by the length of previous anti-VEGFs treatment (*r* = −0.84, *P* < 0.01) and marginally by the presence of SRF before aflibercept initiation (*r* = −0.71, *P* < 0.05).

### 3.2. Anatomic Outcomes

**Figure 2:**
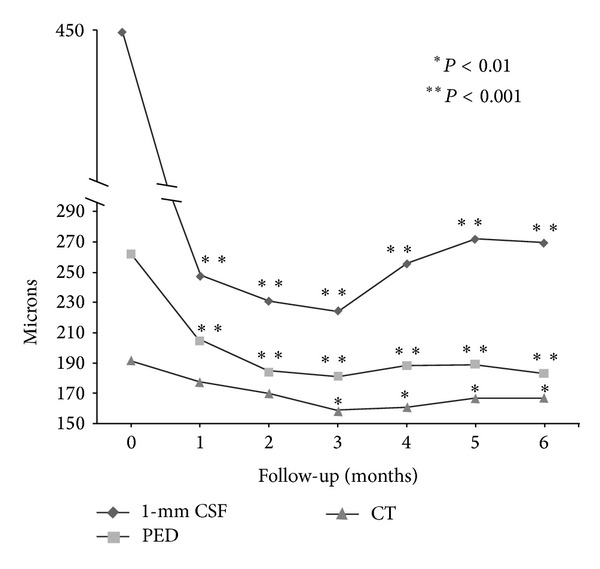
Anatomical results over time after aflibercept conversion. 1-mm CSF: central subfield retinal thickness; PED: pigment epithelial detachment; CT: choroidal thickness.

**Figure 3:**
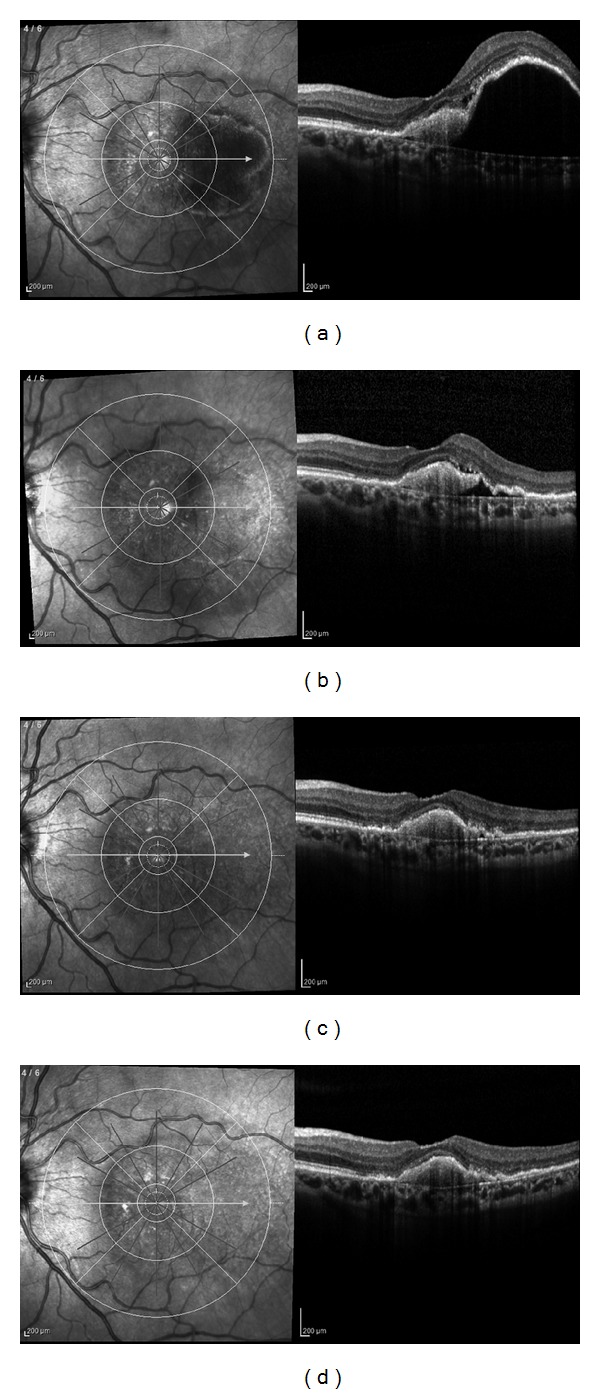
Spectral domain OCT over time of a 67-year-old female previously treated with 30 ranibizumab injections. (a) OCT image 34 days after the last ranibizumab injection showing a primarily hyporeflective PED with a minimal amount of subretinal fluid. Note the complete disruption of the subfoveal IS/OS line. Visual acuity is 35 ETDRS letters (0.40 logMAR). (b) OCT image at month 1, after the first aflibercept injection, shows a decrease in PED height, almost complete resolution of the hyporeflective component of the PED, and a decrease in subretinal fluid. Visual acuity is unchanged. (c) OCT image at month 3, after three aflibercept injections, shows a complete resolution of the hyporeflective component of the PED while the hyperreflective component is almost unchanged. Visual acuity is 33 letters (0.44 logMAR). (d) OCT image at month 6 shows no significant anatomical changes as compared with (c). Visual acuity is 36 letters (0.38 logMAR).

**Figure 4:**
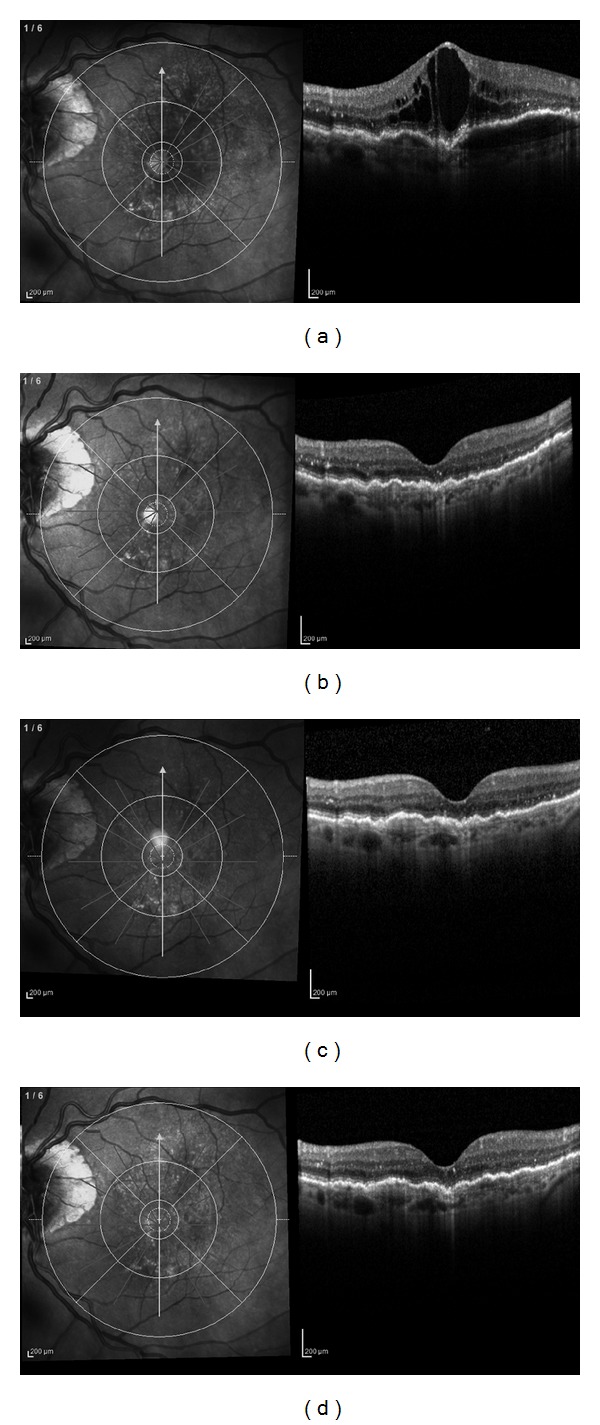
Spectral domain OCT over time of a 73-year-old female previously treated with 12 bevacizumab intravitreal injections and 26 ranibizumab injections. (a) OCT image 37 days after the last ranibizumab injection showing a primarily hyporeflective PED with chronic intraretinal cystic changes. Note the complete disruption of the subfoveal IS/OS line. Visual acuity is 42 ETDRS letters (0.26 logMAR). (b) OCT image at month 1, after the first aflibercept injection, shows a fluid free macula and the complete resolution of the PED. Visual acuity is 40 letters (0.30 logMAR). (c) OCT image at month 3, after three aflibercept injections, shows a fluid free macula and trace of a hyperreflective PED. Visual acuity is 41 letters (0.28 logMAR). (d) OCT image at month 6 shows no significant anatomical change as compared with (c). Visual acuity is 45 letters (0.20 logMAR).

Anatomic results over time are summarized in Table 2. The mean baseline 1-mm CSF retinalthickness was 449 ± 179 *μ*m. After 3 aflibercept injections, CSF retinal thickness decreased significantly to 224 ± 131 *μ*m (*P* < 0.001) and to 269 ± 145 *μ*m at 6 months (*P* < 0.001). Average PED height decreased significantly from 262 ± 134 *μ*m to 181 ± 118 *μ*m (*P* < 0.001) at 3 months and to 183 ± 100 *μ*m (*P* < 0.001) at 6 months. Mean subfoveal choroidal thickness decreased from 192 ± 67 *μ*m to 159 ± 58 *μ*m (*P* < 0.01) after 3 aflibercept injections and to 167 ± 51 *μ*m (*P* < 0.01) at 6 months (Figure 2). Choroidal thickness at 6 months was negatively correlated with the number of aflibercept injections (*r* = −0.85, *P* < 0.01). At baseline, IRF was present in 27 eyes (87%), SRF in 19 eyes (61%), and PED in 31 eyes (100%). After 3 aflibercept injections, 8 eyes (26%, *P* < 0.0001) had IRF, 8 eyes had SRF (26%, *P* < 0.001), and 23 eyes (74%, *P* < 0.01) had PED. At 6 months, IRF was present in 12 eyes (39%, *P* < 0.0001), SRF was present in 8 eyes (26%, *P* < 0.001), and PED was present in 27 eyes (87%, *P* > 0.05). Before aflibercept initiation, 8 eyes (26%) had hollow PEDs, 7 eyes (22%) had solid PEDs, and 16 eyes (52%) had mixed PEDs. At 6 months after aflibercept, mixed PEDs were present in 8 eyes (30%) and solid PEDs in the remaining 19 eyes (70%). Representative cases of treatment response to aflibercept injections are shown in Figures 3 and 4.

None of the studied eyes developed significant ocular adverse events, such as endophthalmitis, acute sterile inflammation, retinal tears, retinal detachment, vitreous hemorrhage, RPE tear, or sustained elevation in intraocular pressure. No systemic thromboembolic events or deaths attributable to the medication occurred. No hypertension was newly diagnosed during the study.

## 4. Discussion 

In the current noncomparative, retrospective study, we evaluated the functional and anatomic response of patients with refractory neovascular AMD after conversion to aflibercept injection. Our results at 6 months showed that aflibercept treated eyes maintained stable visual acuity (VA) and had significant anatomical improvement with a mean number of 4.5 injections. Despite a sometimes dramatic anatomical improvement that occurred mainly after the first injection, our patients did not experience a consistentvisual response. Overall, our observations are in line with the majority of previous reports that observed this kind of dissociation between anatomical and functional results after aflibercept treatment [8–13]. The lack of visual improvement in these eyes affected with recalcitrant neovascular AMD may be related to the longstanding retinal damage as a result of persistent intraretinal and subretinal exudation. Indeed, in our results, BCVA outcome was negatively correlated with both the length of previous treatment and the status of the foveal photoreceptors IS/OS junction line on baseline OCT. There is growing evidence that these eyes respond differently to the treatment-naive eyes evaluated in the clinical trials in which both functional and anatomical improvement occurred within the first 3 months. Therefore, in these patients with persistent exudation, one might also expect that continued treatment with aflibercept, beyond the 6-month period, will allow achieving an improvement in visual acuity. Among the published studies about aflibercept conversion, only two reported a significant VA improvement in refractory neovascular AMD [14, 15]. Comparisons are, however, difficult because of differences in the baseline characteristics of the patients (i.e., length of previous treatment, baseline BCVA), the treatment regimen, and the study design.

Consistent with the results of previous reports, aflibercept treated eyes showed a significant improvement of all the anatomical parameters evaluated [8–15]. Anatomical improvement (i.e., retinal thickness decrease, intraretinal and subretinal fluid reduction, and PED height decrease) occurred, for the most part, after the first aflibercept injection with gains maintained at 6 months. This improvement could be related to a suboptimal treatment with the previous drugs. However, the mean injection interval on prior anti-VEGFs therapy in our series (5.1 ± 0.5 weeks) reflects that patients were overall adequately treated before aflibercept initiation. A possible explanation for this prompt and significant anatomical response to aflibercept in these eyes may be the development of tachyphylaxy/tolerance phenomena. Tachyphylaxy/tolerance is characterized by a decrease of response to a repeated treatment over time and is a recognized phenomenon complicating treatment of neovascular AMD with anti-VEGF agents. Structural and ultrastructural alterations of the neovascular membrane, such as fibrosis, changes of lesion type, chronic changes in the photoreceptors and retinal pigment epithelium, increased expression of VEGF due to increased macrophages migration and activation, increased expression of VEGF receptors, have all been described after chronic anti-VEGF blockade, and have been implicated in the development of tachyphylaxy/tolerance phenomena after anti-VEGF therapy. Further, it has been demonstrated that, after intravitreal administration, both systemic and local immune responses to VEGF inhibitors contribute to the formation of neutralizing antibodies to bevacizumab and ranibizumab [5]. By switching to aflibercept, less initial immunogenicity may lead to improving the anatomical response in eyes refractory to the other anti-VEGF agents. Alternatively, aflibercept increased binding affinity to VEGF-A and its ability to target VEGF-B and placental growth factor may account for the favorable anatomical effects in this setting.

In addition to a significant decrease in PED height, a peculiar modification in the PED characteristics of the sub-RPE material on OCT has been observed in our study, showing the dominance of the solid pattern at the end of follow-up (70%) compared with baseline (22%). As it has been shown for previous anti-VEGF agents, it is possible that aflibercept as well affects primarily the serous, exudative, and hemorrhagic components of a PED, but the fibrocellular component does not respond completely to treatment [16]. Indeed, a large proportion of eyes in our series had persistent PED at 6 months.

After aflibercept treatment, we found a significant reduction of choroidal thickness that was correlated with the number of aflibercept injections. Choroidal thinning was observed from the third month of follow-up. As far as we know, this is the first clinical study showing the effect of intravitreal aflibercept on the choroidal thickness in exudative AMD. A recent prospective study by Yamazaki et al. reported significant choroidal thickness reduction in 23 eyes with typical neovascular AMD treated with 3 loading doses of ranibizumab followed by PRN regimen [17]. As in our results, they demonstrated a significant trend toward choroidal thinning from the third month of therapy, which was sustained for 1 year. Current evidence suggests that VEGF, in particular VEGF-A, may have an important role in the physiological regulation of choriocapillaris survival and permeability. Sustained pan-VEGF inhibition through intravitreal anti-VEGF agents use may therefore influence the integrity of the choriocapillaris, which is known to play a key role in AMD. Previous studies on primate eyes have demonstrated reduction in choriocapillaris fenestrations, formation of immune complexes, thrombotic microangiopathy, and RPE cell death after intravitreal bevacizumab administration [18, 19]. In a recent study comparing the effects of intravitreal ranibizumab and aflibercept in monkey eyes, Julien et al. demonstrated haemolysis, microangiopathy, and reduction of choriocapillaris density with both drugs, whereas formation of protein complexes as well as RPE cell death was only observed after aflibercept [20]. The authors speculated that the differences between the two drugs may be related to the Fc domain of aflibercept and/or to its specific binding properties. Hence, further studies are needed to determine and compare the effects of these biologic agents on choroidal vasculature and their clinical implications.

The limitations of the present study are the uncontrolled retrospective design, the small sample size, and the short-term follow-up. The strengths include the relatively homogeneous cohort of eyes selected with strict inclusion and exclusion criteria and treated by the same retina specialists (MM and LI) and the use of the same standardized treatment protocol before and after aflibercept conversion.

## 5. Conclusions

Our results showed that stable VA and anatomical improvement were obtained for up to 6 months after aflibercept conversion in treatment-resistant neovascular AMD. However, choroidal thinning related to treatment was observed. Prospective, comparative studies are warranted to determine the long-term functional and anatomical results, as well as the most appropriate treatment regimen in this particular setting.

## Figures and Tables

**Table 1:** Baseline characteristics before aflibercept initiation.

	Data (range)
Number of eyes	31
Number of patients, M/F	30, 9/21
Age (years)	70.1 ± 8.1 (60 to 86)
Length of previous anti-VEGF treatment (months)	41.3 ± 14.2 (15 to 58)
Average number of prior anti-VEGF injections	34.4 ± 11.9 (15 to 50)
Interval between the last anti-VEGF and aflibercept initiation (weeks)	4.9 ± 0.8 (4 to 6)
Status of the foveal IS/OS junction, disrupted/complete (%)	20/11 (65/35)

Values are mean ± SD unless otherwise indicated.

VEGF: vascular endothelial growth factor; IS/OS: inner/outer segment.

**Table 2:** Visual and anatomical outcomes after aflibercept conversion.

Outcomes	Baseline	3 months		6 months	
			*P* value^a^		*P* value^a^
BCVA (number of ETDRS letters)	42.5 ± 12.5	42.3 ± 10.5	>0.05^b^	42.8 ± 10.0	>0.05^b^
BCVA (logMAR)	0.24	0.26		0.24	
1-mm CSF (microns)	449 ± 179	224 ± 131	<0.001^b^	269 ± 145	<0.001^b^
Maximum height of PED (microns)	262 ± 134	181 ± 118	<0.001^b^	183 ± 100	<0.001^b^
Subfoveal choroidal thickness (microns)	192 ± 67	159 ± 58	<0.01^b^	167 ± 51	<0.01^b^
Presence of intraretinal fluid, number (%)	27 (87)	8 (26)	<0.0001^c^	12 (39)	<0.0001^c^
Presence of subretinal fluid, number (%)	19 (61)	8 (26)	<0.001^c^	8 (26)	<0.001^c^
Presence of PED, number (%)	31 (100)	23 (74)	<0.01^c^	27 (87)	>0.05^c^
Number of aflibercept injections				4.5 ± 1.3	

Values are mean ± SD unless otherwise indicated.

BCVA: best-corrected visual acuity; ETDRS: early treatment diabetic retinopathy study; logMAR: logarithm of the minimal angle of resolution; CSF: central subfield retinal thickness; PED: pigment epithelial detachment.

^a^Compared with baseline.

^b^Paired *t*-test.

^c^McNemar's test.
